# Deep Eutectic Solvents for Pretreatment, Extraction, and Catalysis of Biomass and Food Waste

**DOI:** 10.3390/molecules24224012

**Published:** 2019-11-06

**Authors:** Payam Kalhor, Khashayar Ghandi

**Affiliations:** 1 MOE Key Laboratory of Bioorganic Phosphorous Chemistry and Chemical Biology, Department of Chemistry, Tsinghua University, Beijing 100084, China; baiy15@mails.tsinghua.edu.cn; 2 Department of Chemistry, University of Guelph, Guelph, ON N1G 2W1, Canada

**Keywords:** deep eutectic solvent, natural deep eutectic solvent, biomass, food residue, pretreatment, extraction

## Abstract

Valorization of lignocellulosic biomass and food residues to obtain valuable chemicals is essential to the establishment of a sustainable and biobased economy in the modern world. The latest and greenest generation of ionic liquids (ILs) are deep eutectic solvents (DESs) and natural deep eutectic solvents (NADESs); these have shown great promise for various applications and have attracted considerable attention from researchers who seek versatile solvents with pretreatment, extraction, and catalysis capabilities in biomass- and biowaste-to-bioenergy conversion processes. The present work aimed to review the use of DESs and NADESs in the valorization of biomass and biowaste as pretreatment or extraction solvents or catalysis agents.

## 1. Introduction

The growing demand for eco-benign processes has led to the discovery of new green solvents [1]. Some ionic liquids (ILs) and deep eutectic solvents (DESs) can be considered green. ILs are defined as organic salts that are liquid below 100 °C [2,3]. They have attracted considerable attention as green solvents due to their remarkable properties, such as non-flammability, recyclability, non-volatility, low vapor pressure, and high boiling point [2,3,4]. Nevertheless, the hazardous toxicity, high cost, difficult synthesis, low biodegradability, and high water solubility of some ILs [3,4,5] have challenged their “green” aspect and driven researchers to explore alternative solvents. DESs, which were introduced at the beginning of this century [5], are prepared by simply mixing two or three components at appropriate molar ratios to form eutectic mixtures with greatly depressed freezing points relative to their components [4,5,6]. DESs make attractive candidates for green solvents, due to properties like short preparation time, low costs, potentially good biodegradability, and low toxicity [4,5,7]. The cost of producing a DES has been estimated to be 20% of that of an IL [8].

Natural deep eutectic solvents, NADESs, which meet green chemistry objectives and are composed of naturally occurring substances from cellular metabolites [9], are considered to be suitable alternatives for organic solvents, ILs, and even for common DESs [10]. Among a diverse list of applications [4,11,12,13,14,15,16,17,18,19], the use of DESs and NADESs in biofuel [8,20,21,22] and bio-oil [23,24] production, as reaction media or extractive agents [25,26,27,28,29,30,31,32,33,34,35], and as media to tune intermolecular interactions [36] are of special importance to researchers.

In recent years, an increasing effort has been made to decrease the use of fossil fuels by substituting them with green and sustainable alternatives [37,38,39,40,41,42,43,44,45,46,47,48] and thus reducing environmental, economic, and societal problems. On the other hand, the agri-food industry produces large quantities of byproducts that are abandoned and can cause potential environmental issues. Meanwhile, these byproducts could be a remarkable source of valuable compounds like phenolic compounds [25], proteins [27], flavonoids [29], anthocyanins [33], lignin [26], peptides [31], polyphenolic antioxidants [49], chitin [50], etc. Therefore, particular interest has been given to food residue and biomass resources, to solve the environmental issues related to waste product management and to valorize these resources to produce green fuels and platform chemicals. All these value-added materials are either obtained via extraction processes or produced by biowaste transformation. In this aspect, the use of DESs and NADESs instead of common organic solvents have drawn significant attention, as they can play essential roles in most of these biochemical processes [51,52,53,54].

**Figure 1:**
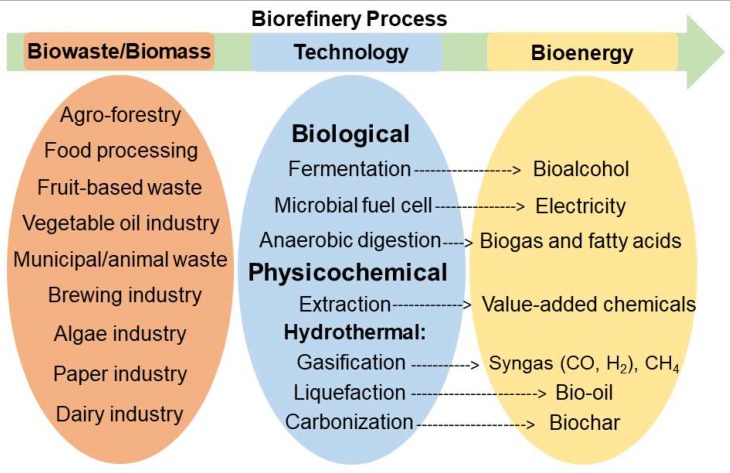
Illustration of the overall biorefinery process to produce bioenergy from biowaste/biomass.

A United Nations Food and Agriculture Organization 2011 report estimated annual global food waste to be approximately 1.3 billion tons [55]. Canadians waste $27 billion of food every year, half of which occurs at the household level [56]. Industrialization and population growth are responsible for the rapid increase of food waste generation worldwide. The manufacturing, agricultural, and food industries harvest large amounts of residues each year, but these are simply discarded as biowaste. These residues contain carbohydrates (cellulose, starch, and sugars), lignin, lipids, proteins, and oils [42,43,48,57,58,59,60,61,62]. There is a growing awareness that the problematic challenges of waste management, resource depletion, and the loss of valuable and energy-containing waste can all be solved; more efficient use of biowaste will contribute to sustainable development. Food waste and food residue can be converted to bio-oil [41,63,64,65,66,67], biogas [68,69], or biochar [37] with hydrothermal methods. Alternatively, the desirable components can be harvested using an extraction process [27,31,33,49,70]. Food waste can also be processed by anaerobic digestion for biogas production [71,72,73,74,75,76] (Figure 1).

**Figure 2:**
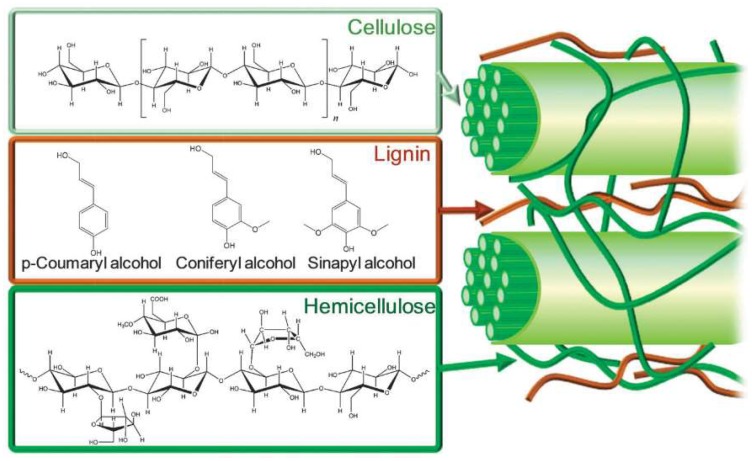
Schematic illustration of lignocellulosic components and their chemical structures. Reprinted from Reference [92] with permission.

Plant-based biomass is a natural, renewable, organic source of carbon [62,77] for conversion to fuel products or valorization to produce biobased chemicals [20,21,26,28,34,35,78,79,80,81,82,83,84,85,86,87]. Biomass conversion into fuels and value-added chemicals decreases the world’s need for fossil fuels and can effectively reduce CO_2_ emissions by combining chemical methods and photosynthesis [88]. The realities of fossil fuel depletion and increasing environmental damage have stimulated both academic and industrial sectors to seek ways to transform biomass. Of all the types of biomass feedstock, lignocellulosic biomass is the most abundant type [89], prevalent in the cell walls of hardwoods, softwoods, energy crops, and other plants [90,91]. The primary constituents of lignocellulosic biomass are carbohydrate polymers such as cellulose and hemicellulose, embedded in a lignin matrix, as illustrated in Figure 2.

**Figure 3:**
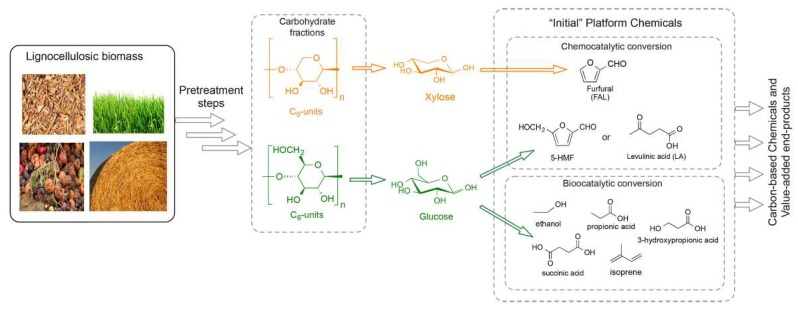
The platform chemicals derived from lignocellulosic components after pretreatment. Reprinted from Reference [98] with permission.

Two feasible methods for the valorization of lignocellulosic biomass for bioenergy applications are: (1) fermentation of sugars from cellulose and hemicellulose components to biofuel [21] or (2) hydrothermal liquefaction and gasification to produce bio-oil [93,94,95] and biogas [96,97] (Figure 1 and Figure 3). In all these methods, pretreatment and solvation are critical steps, and it is important to find green solvents that can substitute the previously used hazardous solvents. DESs and NADESs have captured the attention of the scientific community for their ability to pretreat and selectively dissolve the constituents of biomass (polysaccharides and lignin) or food products (lipids, proteins, and carbohydrates) to yield valuable products.

The present review consists of the following sections: (1) a brief description of DESs and NADESs and their physicochemical properties; (2) recent research on the uses of DESs and NADESs in biomass and food industry processes—these solvents are categorized based on their role in the process (pretreatment solvent, extraction solvent, reaction solvent, or catalyst)—(3) the recyclability of DESs; (4) the effects of the DESs and NADESs on the structure of biomass components. Finally, after a short concluding statement, the future prospects for the possible application of eutectic solvents in the valorization of real food waste in an innovative process design are discussed.

## 2. Definition and Classification of Deep Eutectic Solvents

**Figure 4:**
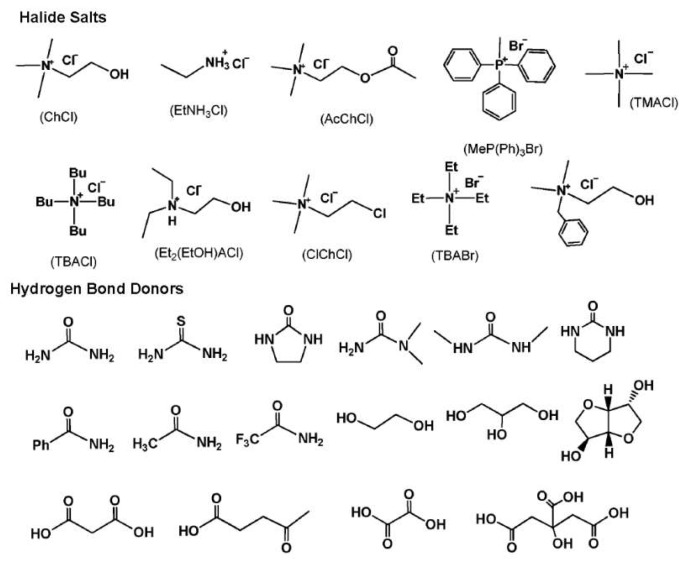
Structures for a number of hydrogen bond acceptors (HBAs) and hydrogen bond donors (HBDs) used for deep eutectic solvent (DES) synthesis.

DESs are eutectic mixtures with their eutectic points lower than that of the ideal liquid mixture [99]. DESs are liquid when they have a eutectic or near-eutectic composition, formed of an appropriately mixed molar ratio of Lewis or Brønsted acids and bases [5,6]. DESs with ionic components are regarded as a new generation of IL analogues, since they have some similarities with ILs. They usually consist of large nonsymmetrical ions, most commonly a quaternary ammonium cation coupled with a halide anion, which is complexed with a metal salt or a hydrogen bond donor (HBD). Figure 4 shows a number of common salts as hydrogen bond acceptors (HBAs) and HBDs used to make DESs.

DESs are classified in Table 1 based on the nature of their HBDs. Type I DESs are made up of nonhydrated metal halide, MCl_x_, and quaternary ammonium salt, Cat^+^X^−^, in the general form of Cat^+^X^−^zMCl_x_ where X^−^ is a Lewis base (x and z refer to the number of Cl^−^ and MCl_x_, respectively). However, the number of nonhydrated metal halides appropriate for a low melting point mixture is limited. Type II DESs are made of hydrated metal halides, MCl_x_.yH_2_O, combined with salts (y refers to the number of H_2_O molecules). Type III DESs typically contain a combination of choline chloride (ChCl) and HBDs such as alcohols, amides, and carboxylic acids. Appropriate HBDs can be mixed with suitable metal halides to form Type IV DESs. For example, ZnCl_2_ suitably mixed with several HBDs, including ethylene glycol, urea, acetamide, and 1,6-hexandiol has been reported by Abbott et al. to form eutectic mixtures [100]. Finally, non-ionic compounds can be used to make mixtures with decreased freezing points to establish a new class, type V, of DESs [101].

### Natural Deep Eutectic Solvents

**Figure 5:**
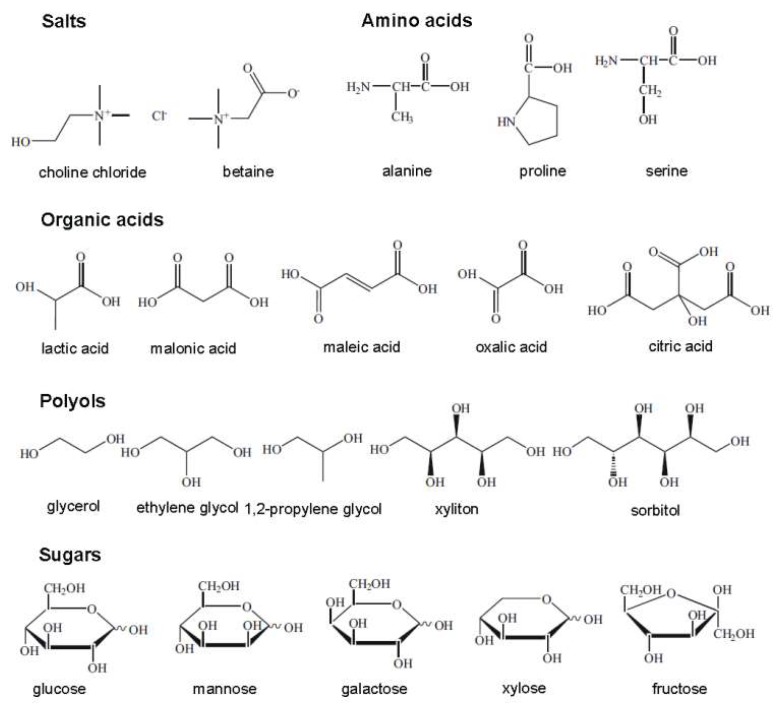
Typical natural constituents used for natural deep eutectic solvent (NADES) synthesis.

The term “natural deep eutectic solvent”, NADES, was proposed to represent mixtures formed by cellular metabolites such as alcohols, amino acids, organic acids, and sugars [9], as shown in Figure 5. They are ubiquitous in nature and are highly applicable because of their superiority over ILs and DESs as being more nontoxic, sustainable, and environmentally benign [102]. In the same way as for a DES, a NADES is obtained by combining HBDs and HBAs in appropriate molar ratios to develop interspecies H-bonds, causing a significant melting point drop. NADESs play major roles in cellular metabolism; many biological phenomena can be explained when considering their formation and existence. For example, many water-insoluble metabolites are transferred into plants because of the presence of such natural solvents. Plants can also survive extremely cold temperatures since the membranes, enzymes, and metabolites are stabilized in plant cells rich in NADESs [103]. In the following section, the physicochemical properties of DESs are discussed. In most cases, the discussion also holds true for NADESs.

## 3. General Information on Deep Eutectic Solvents

### 3.1. Deep Eutectic Solvent Preparation

For preparation of DESs, no solvent is needed and, as no side product forms—except for some ChCl:Carboxylic-acid-based DESs which are discussed in the following section—there is no need for purification of the final product. Most eutectic mixtures are prepared simply by mixing suitably measured components and then stirring at around 80 °C. However, highly viscous sugar-based DESs are difficult to stir. This problem can be overcome by adding extra water into the mixture [104]. Other methods for DES preparation and purification (e.g., removal of water or gases) include a freeze-drying method [105], grinding in a mortar [106], or mixing in an extruder [107]. For example, Gutierrez et al. [105] obtained pure {ChCl:Urea} and {ChCl:Thiourea} DESs in 1:2 molar ratio by dissolving urea (thiourea) and ChCl separately in water in appropriate concentrations and then mixing the mixtures together. Finally, the mixtures were freeze dried to obtain pure DESs.

### 3.2. Physicochemical Properties of Deep Eutectic Solvents

The interest in employing DESs for the biomass and food industries has led to the need for accurate and reliable knowledge of their physicochemical properties. A very important solvent property of DESs is their potential to be tailored. They are task-specific, i.e., they can be tailored to a specific type of chemistry. Compared to ILs, DESs have some superior characteristics: they are recyclable and made of relatively inexpensive components. Many researchers have been intrigued by DESs’ versatile capabilities and have attempted to characterize their physicochemical properties [4,5]. In this section, the main physicochemical features of DESs are discussed.

#### 3.2.1. Thermal Behavior and Phase Diagram

**Figure 6:**
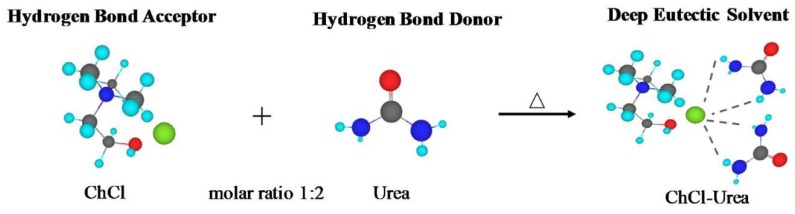
Interaction of HBDs (urea) with HBA (chloride) to form multiple H-bonds. Reprinted from Reference [115] with permission.

**Figure 7:**
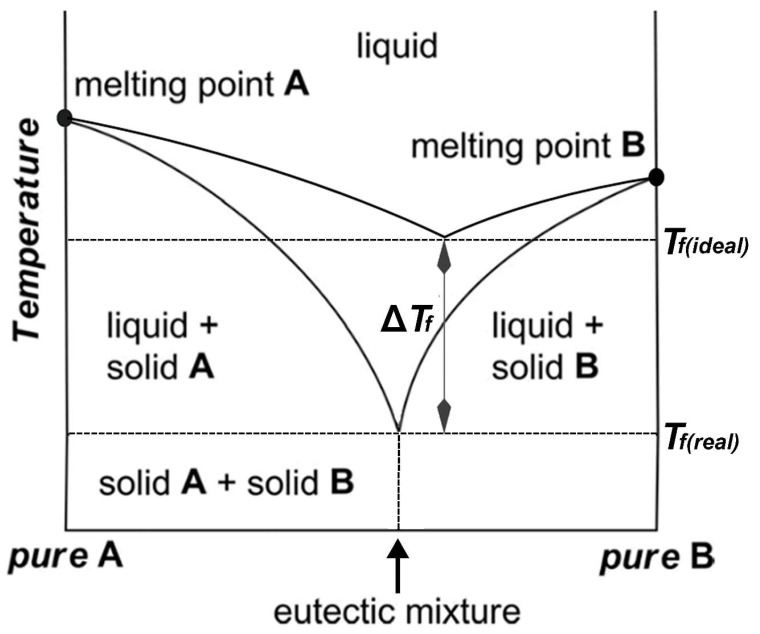
The phase diagram of a two-component mixture representing the eutectic point. *T_f(real)_* is the measured freezing point of a mixture at the eutectic composition and *T_f(ideal)_* is the theoretically predicted freezing point for an ideal mixture.

The thermal behavior and stability of DESs is dependent on their ingredients and molar ratios. Processes for food residues and biomass such as pretreatment, dissolution, or extraction in high temperatures or hydrothermal conditions [23,24,26,27,31,81,108,109,110,111] can only be optimized by understanding DES thermal behavior. A study of the thermal stability of eutectic mixtures based on urea and alcohols or carbohydrates found that the investigated DESs decomposed after heating for 7 h at 80 °C and produced carbonates and ammonia [112]. In another study, it was shown that a series of DESs composed of ChCl and carboxylic acids (glutaric acid, glycolic acid, malonic acid, oxalic acid, and levulinic acid) decomposed in the temperature ranges between 400–500 K [106]. Chemical reactions such as esterification between DES components have also been reported for some ChCl:Carboxylic-acid-based DESs [113]. Accordingly, it was found that a number of DESs containing ChCl and some carboxylic acids (lactic acid, glutaric acid, glycolic acid, malic acid, malonic acid, oxalic acid, and levulinic acid) undergo an esterification reaction between the hydroxyl group of choline and the carboxylic acid [113]. In these cases, esterification reactions take place independent of the preparation method and temperature. In general, many of the physicochemical properties of DESs are influenced by the underlying interspecies interactions, most importantly the HBD–HBA H-bonds (Figure 6). The magnitude of such interactions affects the freezing point depression, ∆*T_f_*, which is defined as:

Δ*T_f_ = T_f(real)_* − *T_f(ideal)_* (1)

where *T_f(real)_* is the measured freezing point of a mixture at the eutectic composition and *T_f(ideal)_* is the theoretically predicted freezing point for an ideal mixture (Figure 7) [99]. These interactions are more favored energetically compared to the interactions that are behind the lattice energies of the pure components [114]. For example, the melting point of a 1:2 molar ratio of {ChCl:Urea} is 12 °C, which is much lower than those of ChCl, 302 °C, and urea, 133 °C [6].

It has been widely assumed, although not universally agreed upon, that the significant melting point depression of a eutectic mixture compared to that of the pure materials is due to charge delocalization from, for instance, the halide anion (HBA) to the HBD, facilitated by hydrogen bond (H-bond) formation [116] (Figure 6). However, the increased strength of the H-bonds developed at a eutectic composition must be counterbalanced by a reduction in strength of several other cohesive interactions [117]. Other decisive factors influencing the melting point values are the lattice energies of the HBA and HBD, the way the anion and HBD interact, and the entropy changes upon DES formation [116]. Abbott et al. suggested that the charge transfer via H-bonds between urea and chloride anion from the ChCl is the main reason of the large melting point depression of the {ChCl:urea} eutectic system at a 1:2 molar ratio [6].

The strength of the H-bond developed between the HBD and HBA can be correlated with the temperature of phase transition, i.e., the stronger the H-bond, the deeper the reduction in melting point [6]. Perkins et al. [118] studied the deep eutectic solvent {ChCl:Urea} system. At the eutectic composition (1:2 molar ratio), the IR spectrum revealed no detectable bands assignable to non–H–bonded N–H, O–H, or C=O groups. It may provide an indication of a specific packing of the system to maximize the intermolecular H-bonds between different moieties. To investigate the molecular interactions, charge transfer, and thermodynamic changes and to find a way to rationalize the freezing point depression, three popular DESs, i.e., reline {ChCl:Urea} and ethaline {ChCl:Ethylene glycol} in a 1:2 molar ratio and maloline {ChCl:Malonic acid} in a 1:1 molar ratio, with freezing points of 12, −66, and 10 °C were selected and studied by Wagle et al. [119]. They characterized different types of H-bonds, such as C–H⋯O and C–H⋯π, as well as conventional O–H⋯Cl^−^ or N–H⋯Cl^−^ interactions which contributed to the DESs stabilization. They showed that charge is mainly transferred from Cl^−^ and Ch^+^ to HBDs. Bond order (BO) analysis revealed that the sum of BOs between Ch^+^ and Cl^−^ in DESs is proportional to freezing point of the DESs. The direct correlation between sum of BOs and charge transfer of Ch^+^⋯Cl^−^ interactions and their straight relationship with the freezing point of DESs clearly demonstrates how the selection of HBDs can affect the physical properties of DESs, such as their freezing temperature. Some mixtures have also been reported to have two eutectic points [120,121]. For example, Cerajewski et al. [120] studied {1-ethyl-3-methylimidazolium Cl:urea} mixtures at different molar ratios by employing electron paramagnetic resonance, differential scanning calorimetry, molecular dynamics simulations, and Raman spectroscopy. They found two eutectic points at 25% and 72.5% urea. It was shown that Cl^−^ is the central species to form networks of H-bonds, in such a way that its quantity determines the extent of intermolecular interactions and the melting behavior of the mixture. They concluded that the macroscopic features of the DES are governed by the nanointerface of the constituents. A direct relationship between features of H-bond network on the basis of topological analysis and the melting point of 45 DESs was established by Garcia et al. [122]. The electron density characterized in the cage critical points (CCP) of the entire DES complex was regarded as a characteristic able to explain the melting point of DESs. They found that lower electron densities in CCPs, which are due to charge delocalization, result in lower melting points. Zahn et al. [123] challenged the well–known concept that charge transfer from the anion to the HBD is responsible for DES formation and the respective depression in melting point. In their communication, they studied three DESs: {ChCl:Ethylene glycol}, {ChCl:Oxalic acid} and {ChCl:Urea}. They found that, despite the first two systems, the charge transferred from Cl^−^ to urea in {ChCl:Urea} mixture is negligible and the urea remains uncharged overall. The respective radial distribution functions (RDFs) suggested weaker Cl^−^⋯Urea H-bonds than those between Ch^+^⋯Cl^−^, which clearly explained the lower Cl^−^⋯urea charge transfer. Therefore, they questioned the assumption that charge spread from anion to HBD is responsible for the decrease in the freezing point of a DES.

To lower melting points of eutectic mixtures, Chen et al. [117] devised a ternary mixture composed of ethylammonium bromide (EABr), butylammonium bromide (BABr), and urea in a 0.6:0.6:1 molar ratio with a eutectic melting point of 10 °C, more than 40 °C less than the eutectic temperatures of {EABr:Urea} and {BABr:Urea} mixtures. The prepared ternary mixture possesses the strongest H-bond interactions, which are offset by cohesive interactions such as electrostatic or van der Waals. Weaker cohesive interactions lead to more orientations of species and, in turn, facilitate H-bond formation. Most DESs have melting points below 100 °C, and a limited number of them are liquid at room temperature. Table 2 lists the melting point of selected ChCl-based DESs. Generally, the lower the melting point, the greater the applicability of the DESs. Accordingly, the DESs with melting points less than 50 °C are more favorable for practical purposes.

#### 3.2.2. Density

The significance of density in designing chemical processes is well recognized [134]. Table 2 gives the densities of some ChCl-based DESs. In general, the reported densities in literature are in 0.785–1.63 g·cm^−3^ range, with the majority falling in the 1.0–1.35 g·cm^−3^ range at room temperature [4,5,135,136], higher than water. The highest density reported is associated with Type IV DESs of a {ZnCl_2_:Urea} mixture at a 1:3.5 molar ratio (1.63 gcm^−3^) [4]. This remarkable difference in densities can be ascribed to molecular packing diversities. However, discrepancies can be found in the literature over the reported densities for a specific DES. For instance, differences up to 4% between density values for {ChCl:Urea} can be found in the available literature [136].

#### 3.2.3. Viscosity

The viscosity of DESs has also been a critical parameter for industrial applications. DESs usually have high viscosities, ascribed to their extensive networks of H-bonds, van der Waals forces, and electrostatic interactions between constituents. Table 2 shows the viscosities of some ChCl-based DESs at different temperatures. The nature of the DES components, their molar ratios, and the temperature are the main factors that determine the viscosity of DESs. Since DESs were introduced as alternative solvents, preparing DESs with low viscosities in order to expand their applicability has been of great interest. It is well known that highly viscous DESs can be made less viscous by adding water. However, because many properties of DESs change remarkably in water presence, DES dilution should be performed with caution. In Section 3.2.4, the effects of water on the physicochemical properties of DESs will be discussed. To date, studies for viscosity measurements have been carried out at atmospheric pressure. More results are needed, especially for viscosity data at elevated pressures. High-pressure conditions are used for the hydrothermal processes used in many biomass and food waste transformations.

#### 3.2.4. Effects of Water

**Figure 8:**
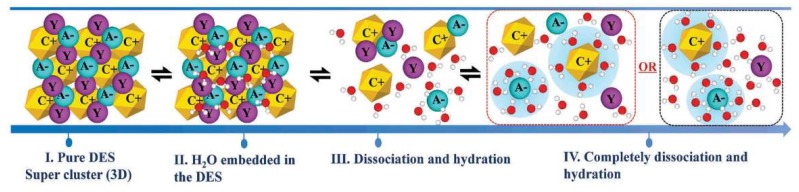
A proposed mechanism for water addition to a typical DES. Reprinted from Reference [139] with permission.

It has been suggested that adding water may decrease the viscosity of DESs and hence increase their usefulness when low viscosity is important [137]. On the other hand, water is usually a main component of biomass and food waste. Most DESs are very hygroscopic and can absorb water from air. Even a trace amount of water influences their structures and properties [138]. DESs contain cations and anions and, therefore, their binary mixtures with water can be very different from ordinary solvents. Figure 8 represents a proposed mechanism for water addition to a typical DES. When water is added to the DES, all the species are hydrated, but it is expected that the anions will be more tightly connected to the surrounding water molecules. Small anions like halides are usually fully solvated, even in highly diluted mixtures [139]. Understanding the effects of water on physicochemical properties of DESs such as density, viscosity, conductivity, surface tension, etc. is crucial. Among all the properties, density and viscosity are the most widely investigated features. The excess values of volume ($V^{E}$) and viscosity ($\eta^{E}$) of DES binary mixtures, which can be either positive, negative, or both, are usually regarded as measures of non-ideality of such mixtures.

##### Effects of Water on the Density of Deep Eutectic Solvents

Density, ρ, and excess molar volumes, $V^{E}$, are often used for analyzing the variations of DESs upon water addition. $V^{E}$ is defined by the following Equation:

(2) $$V^{E}=\frac{x_{1}M_{1}+x_{2}M_{2}}{\rho_{mix}}-\frac{x_{1}M_{1}}{\rho_{1}^{*}}-\frac{x_{2}M_{2}}{\rho_{2}^{*}}$$

where $x_{1}$ and $x_{2}$ are the mole fractions, $M_{1}$ and $M_{2}$ are molecular weights, and $\rho_{1}^{*}$, $\rho_{2}^{*}$ and $\rho_{mix}$ are the densities of Component 1, Component 2, and the binary mixture, respectively. Different DES–water binary mixtures show different $V^{E}$ features throughout the entire compositional range [139,140,141,142,143]. For instance, Kuddushi et al. suggested that the negative $V^{E}$ values of the {ChCl:Malonic acid} and {ChCl:Glutaric acid} DESs combined with water could be due to the dominance of intermolecular interactions, i.e., H-bonds between ions and water and HBD and water over intramolecular interactions [143]. Most of the available experimental data measured the densities of DESs in atmospheric pressure. Only a few studies have investigated the densities or excess molar volumes at elevated pressures [144,145], and these have shown that pressure has a greater effect on $V^{E}$ values than temperature does.

##### Effects of Water on the Viscosity of DESs

Adding water decreases the viscosity of DESs remarkably. To further study the effects of addition of water on DES viscosity, excess viscosity, $\eta^{E}$, can be obtained as follows:

(3) $$\eta^{E}=\eta_{mix}-x_{1}\eta_{1}^{*}-x_{2}\eta_{2}^{*}$$

where $\eta_{1}^{*}$, $\eta_{2}^{*}$, and $\eta_{mix}$ are the viscosity of Component 1, Component 2, and the binary mixture, respectively. The non-ideal behavior of binary mixtures results in a variety of shapes and positive or negative signs of $\eta^{E}$ values [146,147]. All the experimental $\eta^{E}$ results were calculated at ambient pressure, and no data, to the authors’ best knowledge, are available for elevated pressures.

#### 3.2.5. Dielectric Properties

The dielectric properties of mixtures have significant effects on their intermolecular interactions [148]. The static dielectric constant is a measure of the polarity and intermolecular interactions in a solvent [149], which helps to understand the solvation ability of the solvent [150]. The dielectric constants of 42 ILs were measured by Huang et al. [150] using dielectric relaxation spectroscopy. Dielectric relaxation spectroscopy (DRS) is known to be a useful technique to investigate the structural dynamics of liquids [151]. Moreover, for materials of an ionic nature, dielectric spectroscopy is an effective method by which to understand the mechanisms related to charge transport [152]. Despite many studies on ILs, only a few studies on the dielectric properties of DESs have been performed [152,153,154,155,156,157,158,159]. For example, Griffin et al. [159] utilized broadband dielectric spectroscopy in combination with depolarized dynamic light scattering to explore the charge transport and structural dynamics of a deep eutectic mixture, {Lidocaine:2Decanoic acid}. The dielectric spectra at room temperature showed that the mixture was around 25% ionic. They also showed that at elevated temperatures, the mixture had modest direct current (DC) conductivity. One year later, Mukherjee et al. [158] studied the dielectric relaxation of six different acetamide-based DESs. The electrolytes used to form DESs with acetamide were: LiBr, LiNO_3_, LiCl_4_, NaClO_4_, NaSCN, and KSCN. The measurements revealed that the relaxation parameters were dependent on the nature of the electrolytes. Their results suggest that the dielectric relaxation in DESs are similar to those reported for ILs and electrolyte solutions. The two ionic DESs, {Acetamide:LiNO_3_} and {Acetamide:NaSCN},were further investigated by Tripathy et al. [157] using dielectric relaxation spectroscopy in a relatively wide temperature range (173–373 K). They were able to establish the fundamental of the secondary relaxation process. They found that below the temperature of glass transition, two secondary relaxation processes happened. Not only ionic DESs, but non-ionic DESs were also studied to explore their dielectric properties. Mukherjee et al. [155] performed dielectric relaxation spectroscopy and time-resolved fluorescence to study a polyethylene-glycol-based DES. For this DES, the obtained static dielectric constant was large, even greater than that of many polar solvents. Moreover, as for the ionic acetamide DESs, the non-ionic polyethylene-glycol-based DES has a nanosecond relaxation component. Very recently, Reuter et al. [152] employed dielectric spectroscopy to study the reorientational relaxation dynamics and the charge transfer of three DESs, {ChCl:Ethylene glycol}, {ChCl:Urea} and {ChCl:Glycerol}, all at a 1:2 molar ratio. They found that the ionic translational motions and the reorientational motions were closely coupled.

## 4. Major Applications of Deep Eutectic Solvents and Natural Deep Eutectic Solvents

DESs and NADESs are now viewed as convenient green alternatives to many conventional solvents with vast applications. However, in the following section, we present their use only in biomass and food industry processing.

### 4.1. Application of Deep Eutectic Solvents in Biomass and Food Industry Processing

Table 3 gives a selection of DESs/NADESs used as pretreatment agents, solvents, cosolvents, or catalysts in biomass and food industry processes. In several cases, the aim is to produce biofuel and value-added chemicals. Lignocellulosic biomass is a raw material for fuel and chemical production that is available from various sources. As shown in Figure 2, lignocellulosic biomass is comprised of three major constituents: cellulose, hemicellulose, and lignin. The composition of the biomass varies depending on the origin. However, processing the lignocellulosic biomass and its constituents is hindered by low solubility in aqueous and organic systems.

Therefore, DESs or NADESs are usually used for fractionation and pretreatment of lignocellulosic biomass for further processing and/or to selectively isolate the desired component(s) from the remaining matrix. DESs and NADESs are also widely used for the extraction of desired chemicals from different materials. Food residues contain considerable amounts of proteins, lipids, and carbohydrates. Nevertheless, despite the widely recognized potential of such solvents for the extraction of chemicals and pretreatment of materials, their application in the food industry is still rather unexplored.

#### 4.1.1. Deep Eutectic Solvents and Natural Deep Eutectic Solvents as Pretreatment Solvents

The conversion of food residue or biomass to biofuel usually consists of several consecutive stages. Pretreatment, as a key stage in the bioconversion of biomass and food residue, involves the enzymatic hydrolysis, a feasible method for lignocellulosic biomass that reduces the recalcitrance of the biomass [231,232]. The recalcitrance is mainly due to the lignin component.

**Figure 9:**
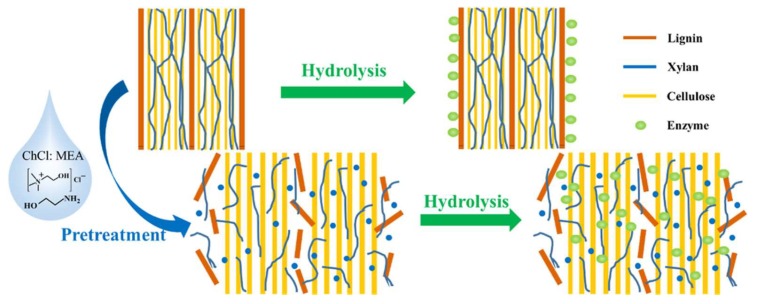
DES pretreatment of wheat straw to reduce the recalcitrance of the biomass for improving enzymatic hydrolysis. Reprinted from Reference [233] with permission.

Figure 9 represents the role of a typical DES as a pretreatment solvent for wheat straw to facilitate the enzymatic hydrolysis of the biomass components.

A pretreatment process can be used to facilitate solvent extraction. Different pretreatment technologies have been developed in the last few decades [234], and each can be employed for a specific objective. At present, these technologies suffer from several drawbacks, most importantly the high cost and harmful effects of the pretreatment agents on the desired components. To overcome these disadvantages, DESs and NADESs have been introduced as green pretreatment agents. They are used on lignocellulosic biomass to improve the production of biofuel. The power of these solvents to dissolve the hard lignin component of biomass has paved the way for biomass pretreatment under mild conditions.

In a study, Xu et al. [8] used seven DESs to pretreat corn stover for obtaining biobutanol. Among all the DESs, the acidic {ChCl:Formic acid} showed the best performance for hemicellulose and lignin removal. Chen at al. [26] used {ChCl:Ethylene glycol} DES under acidic conditions to pretreat switchgrass. Lignin and hemicellulose components were substantially removed and the cellulose was enriched up to 72.6%. The fermentable sugar was finally converted to 90.2 g/L 2,3-butanediol.

Mamilla et al. [164] prepared and applied several ChCl-based DESs to fractionate lignocellulosic biomass. {ChCl:Oxalic acid} and {ChCl:KOH} DESs proved to be more effective in dissolving beech wood polymers. During the experiment, other parameters such as reaction time, temperature, and the chip to solution mass were controlled. The results showed that {ChCl:Oxalic acid} DES separated phenols selectively, and that this DES could be scalable for employment in biorefinery plants where lignin is to be isolated first.

Precentese et al. [169] pretreated apple residues, potato peels, coffee silverskin, and spent brewer’s grains with {ChCl:Glycerol} and {ChCl:Ethylene glycol} DESs to produce fermentable sugar. The optimum operating conditions were 3 h of pretreatment with {ChCl:Glycerol} DES with a solid:solvent ratio of 1:16 at 115 °C.

The fractionation efficiency of lignocellulosic material can be improved when using an additional hydrothermal pretreatment. For instance, in order to increase the efficiency of the {ChCl:Glycerol} DES treatment, a prior hydrothermal pretreatment was applied to reduce the recalcitrance of date palm residues [81]. This proposed approach revived the efficiency of DESs for cellulose digestibility. Liang et al. [166] used hydrothermal and {ChCl:Ethylene glycol} DES pretreatment for biomass fractionation and lignin removal. At the end, they separated and recovered the DES components by electrodialysis with recovery ratios of 92% and 96% for ChCl and ethylene glycol, respectively.

Some researchers used in situ prepared eutectic solvents, with one component usually taken from the biomass. For example, Yu et al. [167] proposed a modified liquid hot water (MLHW) process for the pretreatment and delignification of garden wastes based on in situ preparation of {ChCl:H_2_O} DES. For one type of the tested biomass (leaf sheaths of *Roystonea regia*, LSR), the biomethane yield was improved by as much as 309.0%.

Shen et al. [165] developed a biomass-derived {ChCl:Lactic acid} NADES (1:10 molar ratio) pretreatment to deconstruct eucalyptus structure at 110 °C and 6 h for the removal of hemicellulose and lignin. Under this optimum condition, the glucose yield was up to 94.3%, 9.8 times higher than the original biomass without DES pretreatment.

In some cases, water can be mixed in with the eutectic solvent to, for example, improve the viscosity of the media. However, the new aqueous mixed solvent gains new characteristic features. To study the effect of water addition and consequent improved viscosity of {ChCl:Urea} DES for delignification of oil palm fronds, New et al. [168] pretreated the biomass sample with the prepared aqueous DES at 120 °C for 4 h. They found that the DES/water mixture had an improved lignin removal ability compared to pure DES. The energy requirement for biomass transformations can also be considered. Procentese et al. [21] compared the energy required for pretreatment of lettuce leaves to produce biobutanol by {ChCl:Glycerol} solvent and by NaOH solvent and steam explosion method. They found that DES pretreatment with 94.9% glucose and 75.0% xylose yield, required 28% and 72% less energy than NaOH and steam explosion processes, respectively.

Microalgae-derived lipids are regarded as a sustainable biodiesel feedstock alternative. In order to extract lipids from microalgae biomass using dimethyl carbonate (DMC) and supercritical CO_2_ solvents, ChCl-based DESs combined with microwaves were used for pretreatment [110]. It was found that DESs made of {ChCl:Carboxylic acids} had an increased selectivity (16%) and increased total fatty acid (TFA) extraction yield (80%) in DMC. This pretreatment also improved the extraction yield of lipids in supercritical CO_2_.

**Figure 10:**
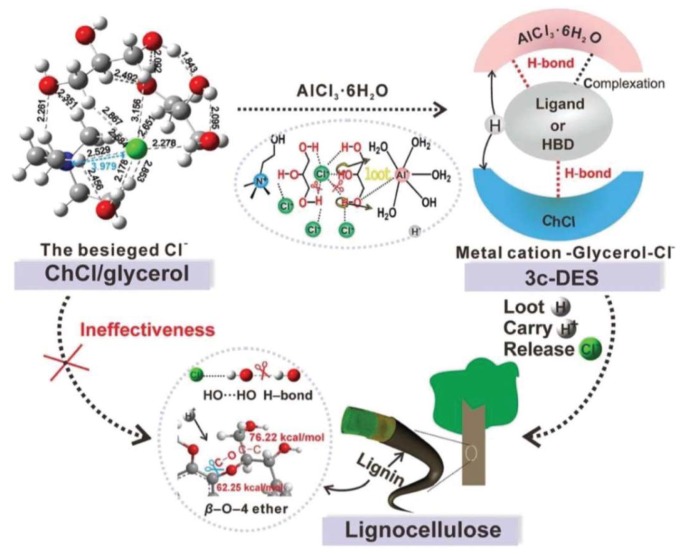
Illustration of the designed ternary DES and its efficiency in biomass fractionation. Reprinted from Reference [80] with permission.

However, not all DESs are always suitable for biomass fractionation and breakage of lignin–carbohydrates. For example, it has been reported that {ChCl:Glycerol} DES is less effective at fractionating lignocellulosic biomass than other types of DES [81,84,203]. Therefore, coordinating suitable components into the DESs and preparing a ternary DES can significantly improve the DES’s power to fraction biomass. In the case of {ChCl:Glycerol} DES, Xia et al. [80] conducted a study using density functional theory (DFT) and Kamlet–Taft solvatochromic methods to analyze the nature of the interactions between the DES components and biomass, and to explore why this DES has low efficiency in lignin fractionation during pretreatment processes. They found that the decreased efficiency of the DES in lignin solubilization (breakage of lignin–carbohydrate linkages) is because of the mutually anionic and cationic H-bonds in the DES network, which result in weak competing interactions towards the biomass linkages. Moreover, as the DES lacks acidic sites, the ether bonds of the biomass are not broken. To increase the DES efficiency, they incorporated AlCl_3_.6H_2_O into the DES to design a ternary DES (Figure 10). The resulting multisite-ligand-containing DES could effectively break the ether bonds as well as the H-bonds of the biomass. By doing so, the lignin fractionation yield increased from 3.61% to 95.46%.

In another study, a ternary DES composed of {guanidine hydrochloride:ethylene glycol:p-toluenesulfonic acid} was developed to pretreat switchgrass and produce concentrated sugar hydrolysate [171]. This solvent was the most efficient DES, with 79% xylan and 82% lignin removal at 120 °C and in 6 min with 10 wt. % solid loading.

#### 4.1.2. Deep Eutectic Solvents and Natural Deep Eutectic Solvents as Extraction Solvents

The number of studies on extraction and separation media for bioactive compounds is increasing. Since DESs and NADESs are composed of simple and naturally occurring components, they can be employed for the extraction of desired compounds such as proteins, peptides, phenolic compounds, etc. from plants or other matrices. In one recent study, six types of DES were employed to extract collagen peptides from cod skins [31]. Based on the criteria of high extraction efficiency and high purity, {ChCl:Oxalic acid} DES was considered to be an optimal solvent for extraction. However, the efficiency was influenced by several factors, including the molar ratio of DES components, the operating temperature, and the ratio of DES to sample.

Shrimp shells are a source of several valuable chemicals that can be extracted by eutectic mixtures. Huang et al. [182] used a NADES, {ChCl:Malic acid}, to extract chitin from shrimp shells. Assisted by microwave irradiation, the NADES could remove most of the minerals and proteins from the shells. In another study, chitin was extracted from shrimp shells by various NADESs to produce chitin films. Among all tested NADESs, {ChCl:Malic acid} NADES extracted the highest purity chitin with a yield of 19.41% ± 1.35%, higher than the conventional method (16.08% ± 0.57%) [50]. The conventional method included treatment of the demineralized sample with 6% HCL (*w*/*v*) and the treatment of the residue with 10% NaOH (*w*/*v*) [212]. Feng et al. [177] used acidic NADESs with decalcification, deproteinization, and acylation abilities. The nature of the NADES, temperature, and time were key factors affecting the experiment efficiency. With {ChCl:DL-malic acid} NADES in a 1:2 molar ratio, the purity of *O*-malate chitin was up to 98.6%.

Microalgae biomass has many bioactive substances. In a study by Mahmood et al. [199] to evaluate the ability of 12 ChCl-based DESs and compare their performance with two benchmark conventional solvents (ethyl acetate and water), microalgae biomass was subjected to extraction for recovering polyphenols at 60 °C, with a DES to biomass ratio of 20:1 for 100 min [199]. The results support the superiority of DESs over conventional solvents for polyphenolic extraction yield.

Agri-food industrial by-products are also subjected to valorization. In a study conducted by Garcia et al. [32] a variety of ChCl-based DESs were used to extract phenolic compounds from virgin olive oils. Two of the DESs, {ChCl:Xyliton} and {ChCl:1,2-Propanediol} showed a profound increase of extraction up to 20%–33% and 67.9%–68.3% compared to a conventional 80% (*v*/*v*) methanol/water. In 2017, Bosiljkov et al. [202] performed an ultrasound-assisted {ChCl:malic acid} NADES extraction of wine lees anthocyanins. In their study, the optimum conditions were: 30.6 min of extraction time, 341.5 W of ultrasound power and 35.4% water content in the NADES (*w*/*w*). Grudniewska et al. used {ChCl:Glycerol} DES for enhanced extraction of proteins from oilseed cakes [27]. First, they extracted the proteins into the DES. The extract was then precipitated upon addition of an antisolvent, water. The noticeable point is that the yield of precipitate increased with increasing temperature of the treatment.

Fernandez et al. [186] employed a novel NADES, {Lactic acid:Glucose}, {Citric acid:Glucose}, and {Citric acid:Fructose} in, respectively, 5:1, 1:1, and 1:1 molar ratios for ultrasound-assisted extraction of 14 phenolic compounds from onion, olive, tomato, and pear byproducts at 40 °C. The aqueous {Lactic acid:Glucose} NADES was selected as the optimal solvent. To show the power of the NADES in the extraction of phenolic compounds, the extraction efficiency of the eutectic solvent was compared to those from methanol and water. It was concluded that the {Lactic acid:Glucose} NADES yielded higher extractability.

Deng et al. [196] synthesized a series of water-soluble DESs composed of hexafluoroisopropanol (HFIP) as HBD and l-carnitine/betaine as HBAs to extract pyrethroid residues from tea beverages and fruit juices. The results indicated that the extraction method based on {l-Carnitine:HFIP} (1:2 molar ratio) solvent had several advantages, such as a short extraction time and high enrichment factor. In 2019, Cao et al. [25] applied a combination of {ChCl:Ethylene glycol} DES (1:3 molar ratio) with a homogenate-assisted vacuum-cavitation extraction method for isolation of phenolic compounds from rattan. Under the optimum conditions, the extraction efficiency of total phenolic compounds was 6.82 mg/g.

Eutectic mixtures can perform as reaction media as well as extraction solvents for bioconversion of a number of components. For example, Gioia et al. [221] explored the possibility of a selective conversion of furfural, produced by biomass, to bifunctionalized cyclopentenone derivatives in a DES made of {ChCl:Urea}. In another study, cellulose derived from sunflower stalks was converted to some value-added components in a DES medium [223]. Three DESs, namely, {ChCl:Oxalic acid}, {ChCl:Citric acid}, and {ChCl:Tartaric acid} were used as solvents and catalysts. With {ChCl:Oxalic acid} DES and under microwave irradiation, 99.07% carbon efficiency was obtained at 180 °C in 1 min. Under such conditions, 4.07% of 5-hydroxymethyl furfural (5-HMF), 76.2% of levulinic acid, 5.57% of furfural, and 15.24% of formic acid were produced.

The applicability of NADESs for the removal of cadmium from rice flour was examined by Huang et al. [210]. Among the ChCl-based and glycerol-based NADESs, the former demonstrated good removal of Cd (51%–96%). The interesting point was that the NADESs did not affect the structure or chemical components of rice flour.

Anaerobic digestion of biological and food wastes produces biogas, which is considered a renewable energy supply. Biogas’ main impurity is CO_2_, which should be removed in the upgrading process. In a very recent study [176], the well-known DES {ChCl:Urea}, in aqueous form, was employed as a liquid absorbent in a conceptual process to upgrade biogas. For the simulation, experimental thermophysical properties were evaluated. In comparison with a pure water process, it was concluded that the DES addition decreased the energy use by 16%. Moreover, to study how environment could be influenced by the process, they employed the Green Degree (GD) assessing method [235]. In this method, an integrated index containing nine environmental factors is reported. The DES loss was negligible due to its very low vapor pressure and thermal stability. They found that the calculated difference of GDs, ΔGD, was higher than zero for aqueous {ChCl:Urea} processes, implying that this process is environmentally benign.

#### 4.1.3. Deep Eutectic Solvents as Catalysts

In spite of their great potential, the use of DESs as catalysts or cosolvents in hydrothermal liquefaction of biomass has barely been studied. In a study in 2016 [24], the efficiencies of four ChCl-based DESs as catalysts and cosolvents ({ChCl:KOH}, {ChCl:p-toluenesulfonic acid monohydrate}, {ChCl:Glycerol} and {ChCl:Ferric Cl}) and their effects on biocrude production were evaluated in a hydrothermal liquefaction process of de-oiled Jatropha cake. The highest biocrude yield was obtained by {ChCl:KOH} (43.53%) and {ChCl:*p*-Toluenesulfonic acid monohydrate} (38.31%) DESs. They found that when using DESs as catalysts and cosolvents simultaneously in the hydrothermal liquefaction of biomass, the selectivity of biocrude could be increased. They also found that DESs containing HBDs preferred to yield aromatic oil via the condensation and hydrolysis of lipids. In another study, {ChCl:*p*-Toluenesulphonic acid} DES was employed as a catalyst for the co-liquefaction of glycerol and whole *Jatropha curcas* seed to obtain bio-oil [23]. Among all the parameters, temperature was found to be the predominant one. The results were compared with those of Na_2_CO_3_-catalyzed liquefaction. The bio-oil yield and oxygen content were higher for liquefaction (32.87% and 28.15 ± 0.88 wt. %) than co-liquefaction (8.99% and 21.58 ± 0.70 wt. %). The higher heating value (HHV) of bio-oil from co-liquefaction (31.73 ± 0.69 MJ/kg) was higher than that of liquefaction (28.80 ± 1.32 MJ/kg). Therefore, it was concluded that the bio-oil yield was decreased with the DES-catalyzed process, but the quality of the product improved. Machmudah et al. [109] extracted xanthone and phenolic compounds from pericarps of mangosteen. They employed a subcritical water treatment, containing deionized water and 10%–30% (*v*/*v*) {Citric acid:Analine} DES as extraction media. Different temperatures (120–160 °C) and pressures (1–10 MPa) in batch and semi-batch systems were applied. They concluded that the higher extraction efficiency in presence of the DES seemed to be due to the role of the DES as a catalyst in the solution. To produce biodiesel from non-edible seed oil, {ChCl:*p*-Toluenesulfonic acid} DES was synthesized to be used as heterogeneous (supported on silica gel) and homogeneous (without support) catalysts. In the temperature range 273–353 K, the catalysts showed thermal stability. The homogeneous catalyst had a dual role of catalyst and cosolvent, reducing reaction time and enhancing the phase homogeneity. The homogeneous and heterogeneous catalysts were recycled for four and seven cycles, respectively. It was concluded that both types of catalysts effectively produced an acceptable quality biodiesel through a single step esterification [230].

**Figure 11:**
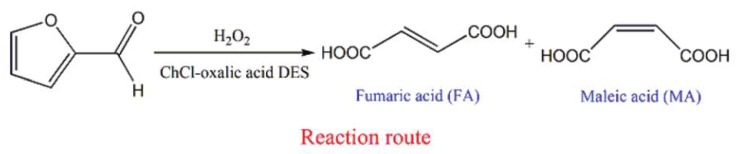
Fumaric acid and maleic acid obtained from furfural in {ChCl:oxalic acid} DES. Reprinted fromReference [229] with permission.

Under some conditions, a component of the DES can perform as a catalyst in a bioconversion. For instance, in a very recent study, Ni et al. [229] explored the conversion of biomass-derived furfural to maleic acid and fumaric acid with {ChCl:Oxalic acid} DES, where oxalic acid worked as an acidic catalyst for the mentioned conversion (Figure 11). The maleic acid and fumaric acid conversion yield 95.7% at 50 °C with H_2_O_2_ as an oxidizer.

## 5. Recyclability of Deep Eutectic Solvents

Recyclability of a solvent is desirable to achieve an economically and environmentally sustainable material extraction or pretreatment process. This usually involves recovery or separation and, if necessary, purification of the solvent, followed by reusing or recycling it. When it comes to eutectic solvents, besides pretreatment or extraction efficacy, recyclability is a significant advantage [52,79,236]. The number of cycles for DES regeneration has so far been limited by factors such as a decrease of performance efficiency, thermal instability, and susceptibility to contaminants [237,238]. Kumar et al. [239] reported no loss in performance strength of used DES after three cycles of pretreatment. Gioia et al. [221] used {ChCl:Urea} as a reaction medium and claimed that the solvent could be reused up to four times with no reduction in its efficiency.

**Figure 12:**
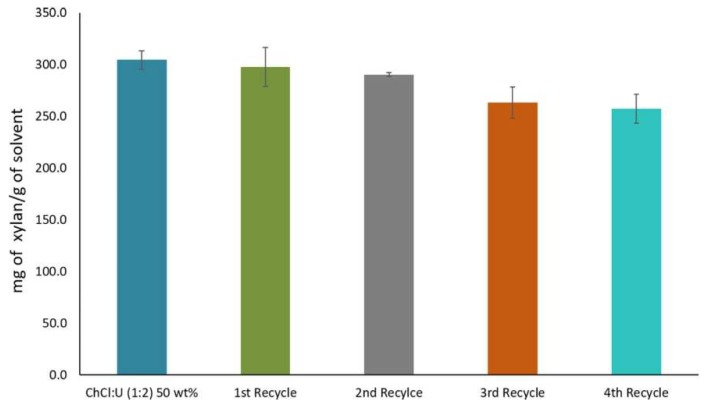
Solubility of xylan and recyclability yield of aqueous {ChCl:urea} DES. Reprinted from Reference [28] with permission.

However, reduction in recycled DES efficiency has been observed in some cases. Shen et al. [165] found that the recovered yield of {ChCl:Lactic acid} DES, evaluated by mass measurement was at least 90% per cycle. In a study by Morais et al. [28], xylan was solubilized and extracted by aqueous {ChCl:Urea} DES. The DES was then successfully recycled up to four times. However, as shown in Figure 12, xylan solubility decreased by 5% after the first two cycles. The decrease in the DES efficiency was due to the dissolution of some low-molecular-weight compounds like furfural and phenolic compounds that did not precipitate from the DES through the process.

**Figure 13:**
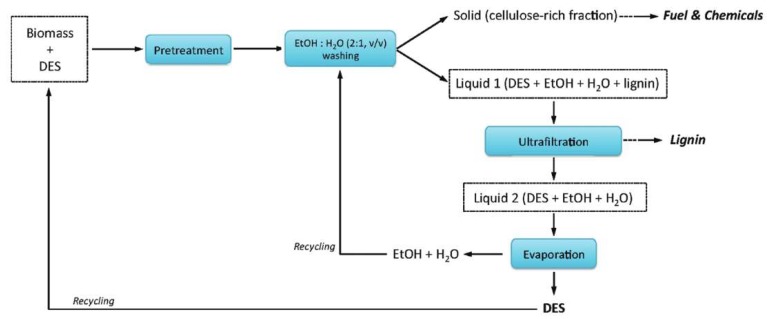
Flow diagram showing a simplified process involving DES recycling. Reprinted from Reference [79] with permission.

The DESs can be recovered with different methods [79,240,241,242,243,244]. Ultrafiltration is a common method. For example, in the example illustrated in Figure 13 for a pretreatment process of a typical biomass (switchgrass) [79], Liquid 1 which contains DES, extracted component (lignin) and cosolvents (water and ethanol) is separated from the solid residue. The dissolved lignin is then separated from Liquid 1 by ultrafiltration. Finally, Liquid 2 is heated to evaporate the cosolvents to obtain pure DES.

The recovered DESs are sometimes examined to analyze any possible structural changes to their components during the process. For example, FTIR was used to qualitatively analyze the recovered ChCl and ethylene glycol components of the {ChCl:Ethylene glycol} DES after a combined hydrothermal and DES pretreatment [166]. For both components, no significant change was observed in the stretching or bending vibrations of O–H and C–H. The structural maintenance of components indicated the efficient recovery method (ultrafiltration and electrodialysis) in the bioconversion process.

## 6. Effects of Deep Eutectic Solvents and NADESs on the structure of biomass components

Different components of the biomass and food residue are chemically influenced by solvents during any specific stage of bioconversion. Depending on the type of the DES and other conditions such as temperature, pressure, and pH of the mixture, the structures of these components may change. For extraction purposes, structural modifications of the isolated species are highly avoided. There are varieties of experimental techniques with which the structural changes of target constituents are revealed upon solvent addition and through the process. Among the experimental methods, XRD and SEM analysis, and FTIR, UV-vis, and NMR spectroscopies are of high importance for structural exploration. Additionally, the use of such techniques may help to identify and prove the existence of the desired components and to study the extraction mechanism.

In a study, delignification of corncob was performed with three ChCl-based DESs as pretreatment solvents [22]. XRD, FTIR, and SEM were employed to explore the structure of the sample during the process. The XRD experiment revealed that the crystallinity index of corncob residues had a minor increase upon DES pretreatment. Because the crystallinity index of cellulose considerably decreased after the same pretreatment process, it was concluded that the relative amount of cellulose in corncob residues increased due to hemicellulose and lignin removal. The SEM images also indicated that the surface of the corncob was roughened and disordered after being pretreated. This was attributed to destructuration of the corncob by DESs via lignin and cellulose removal. In the FTIR analysis, the decrease in the amplitude of the wavenumber bands assigned to H-bonded hydroxyls in cellulose after DES addition indicated the formation of stronger H-bonds between DESs and corncob. The intensity decrease and disappearance of the band at 1737 cm^−1^ after pretreatment by {ChCl:Carboxylic acid} and {ChCl:Polyalcohol} was ascribed to the rupture of the ether bonds between hemicellulose and lignin. Furthermore, the decrease of the band at 834 cm^−1^ after pretreatment was indicative of delignification. In another study, FTIR and NMR spectroscopies were used to explore the molecular structure of lignin, isolated from wheat straw biomass, before and after pretreatment with {ChCl:ZnCl_2_} at a 1:2 molar ratio [245]. The FTIR results indicated that the backbone structure of lignin did not change much after DES pretreatment. However, the phenolic hydroxyl in the precipitates increased as the carbonyl groups decreased. The ^13^C-NMR analysis also suggested that the DES used had little impact on the amount of aromatic ring substitution.

In another study, a 2D NMR experiment on the lignin extracted from switchgrass via {ChCl:Ethylene glycol} pretreatment revealed the cleavage of *β*-O-4 linkages in lignin, which facilitated the solubilization of lignin. This clearly showed the importance of the acidic protons in the DES [26]. Huang et al. [182] employed several techniques, namely FTIR, XRD, and TGA, to explore the chemical composition changes of the extracted chitin from shrimp shells using {ChCl:Malic acid} NADES and acidic/alkaline solvents. Regarding FTIR spectroscopy, they found that the spectra of the shrimp shells was considerably different from those obtained from NADES-/acid-/alkali-extracted chitin. The XRD of NADES-extracted samples showed a crystal lattice type of α–chitin. The increase in crystallinity index indicated that mineral and proteins were extracted from shells by NADES. For the TGA experiment, in the range of 200 to 250 °C (the range typically observed for proteins in shrimp shells) the mass loss was absent in the NADES-extracted chitin, indicating that the proteins were removed by NADES.

**Figure 14:**
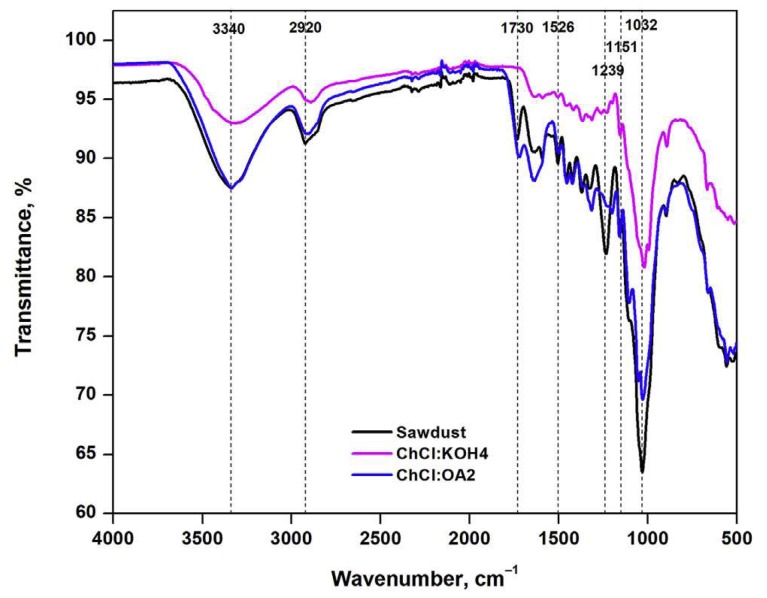
FT-IR spectra of the lignocellulosic biomass samples before and after DESs pretreatment. Reprinted from Reference [164] with permission.

A series of strongly basic DESs was used to pretreat wheat straw for delignification [233]. XRD analysis of the sample was carried out before and after DES pretreatment. The results of the untreated sample showed that its crystalline structure was the native cellulose I crystal type. As the pretreated sample did not reveal any alteration in crystal type, it was concluded that the DESs that were used could not disrupt the crystalline structure of the wheat straw. However, the crystallinity index suggested higher crystallinity of the sample after DES pretreatment. The IR analysis indicated decreases in the characteristic bands of DES-pretreated wheat straw compared to untreated samples. This implies the depolymerization of lignin and hemicellulose via pretreatment. Very recently, FTIR spectroscopy was used to study the structural modifications of the used lignocellulosic biomass, beech wood polymers upon DES ({ChCl:KOH} and {ChCl:Oxalic acid}) pretreatment. As illustrated in Figure 14, the size of the peaks at 990, 1032, and 1160 cm^−1^ assigned to C–O, C=C, C–C–O, and C–O–C groups of cellulose, were reduced when pretreated by {ChCl:KOH}, confirming the cellulose removal from the biomass sample. Furthermore, the disappearance of the two peaks at 1215 and 1270 cm^−1^, attributed to C–C and C–O vibrations of the lignin aromatic ring, indicated lignin removal from biomass upon {ChCl:Oxalic acid} pretreatment. Considering the hydroxyl stretching region, the peak of lignin is usually wider and that of cellulose is deeper. In Figure 14, the peak at around 3340 cm^−1^ is broader for {ChCl:KOH} and deeper for {ChCl:Oxalic acid} DESs, indicating that the hydroxyls were from phenols (lignin) and cellulose molecules, respectively.

The FTIR and UV-vis spectroscopies were examined for collagen peptides extracted with DESs from cod skins [31]. The strong absorbance at 218 nm of the collagen peptides in {ChCl:Ethylene glycol} DES was assigned to n→π* transitions of carbonyl in the peptide bonds, indicating that neither of the DESs affected the peptide structure, or any chemical bond formed between peptide and the DES. However, the other DES, {ChCl:Oxalic acid}, behaved differently. The IR spectra showed that the bands assigned to collagen peptides disappeared when peptides were dissolved in {ChCl:Oxalic acid} DES, meaning that the functional groups of peptides were broken under the effect of this acidic DES. Grudniewska et al. [27] obtained the solid state ^13^C-NMR, FTIR, and TGA of oil cakes (RC and EC), biomass residues (RCBR and ECBR), and precipitates (RCP and ECP) after biomass pretreatment with {ChCl:Glycerol} to investigate any structural change of the biomass and characterize the proteins in the precipitates.

Regarding only the ^13^C-NMR analysis, the spectra revealed signals of cellulose and other structural polysaccharides for the oil cakes and biomass residues. The spectra also signified a broad peak attributed to the carbonyl groups of proteins, hemicellulose, and lignin. The intensity of the carbonyl group of the biomass residues, compared to that of oil cakes, decreased and the signal features of cellulose increased. For the precipitants (RCP and ECP), the intensity of carbonyl group increased, while cellulose C1 signal disappeared (ECP) or diminished (RCP). This also happened to polysaccharide sugar units where C2, C4, C5, and C6 signals decreased compared to oil cakes. In both precipitates, the signals at 65–48 ppm and 56–54 ppm were attributed to α-carbons found in the proteins and CH_3_O in lignin.

## 7. Conclusions and Future Prospects

In this article, we reviewed DESs and NADESs in state-of-the-art technologies for biomass/biowaste valorization, where DESs and NADESs were used as reaction media, pretreatment or extraction solvents, catalysts, or as multifunctional solvents. A variety of multipurpose eutectic mixtures can be prepared with properties superior to those reported for ILs; the eutectic mixtures can be made to be less toxic, more biodegradable, and quicker and easier to prepare. Their unfavorable properties can be surmounted by tailoring them, for example by changing the nature of the salt or its molar ratio to HBD, adding appropriate cosolvents, or simply by changing temperature or pressure. If the DES or NADES suffers from high viscosity, adding water in measured amounts works well. In biomass and food waste valorization, materials can be pretreated to enhance enzymatic hydrolysis and selectively solubilize the desired components or catalyze the thermochemical processes. They can also be used as reaction media for chemical and biochemical processes. In some cases, the efficiency of the all the above-mentioned functions of DESs or NADESs could be increased if combined with another technique. For example, the pretreatment power of the solvents improved when coupled with microwave or ultrasonic irradiation or hydrothermal methods. Eutectic solvents can, however, have serious impacts on the structures and functional groups of biomass components.

The existing routes for the bioconversion of biomass and food residue should be optimized, with the possibility of taking full advantage of the features and advantages of eutectic solvents. We looked into the future prospects of the use of DESs and NADESs for valorization of real food waste, and the feasibility of a successive two-step process for biofuel and bio-oil production through sugar fermentation and hydrothermal liquefaction, where DESs and NADESs have the potential to be employed as multifunctional agents. There are three aspects of future study that we think are important.

i. Using real food waste instead of only lignocellulosic biomass, single-component biowaste, or even food waste models for production of chemicals, biofuel, and bio-oil: Food waste can provide free biomass from many sources, including households, restaurants, and food processing industries. There are several methods able to transform biomass, single-component wastes, or multi-food waste into liquid, solid, or gaseous fuels [37,65,68]. However, food waste is seldom used and, to our knowledge, no single study has yet explored the use of DESs or NADESs for such purposes.

ii. A successive two-step process for biofuel and bio-oil production via sugar fermentation and hydrothermal liquefaction: Food waste can be first pretreated and enzymatically hydrolyzed to produce fermentable sugars, after which biofuel is obtained through microbial fermentation. The unhydrolyzed residue usually contains undigested recalcitrant carbohydrates, lipids, and proteins, and can be transferred to hydrothermal reactors for further processing. Hydrothermal processes involve thermochemical conversion of material using high-pressure and high-temperature water to decompose the polymeric material structures. Depending on the type of the hydrothermal analysis, bio-oil, biochar, or biogas is produced by hydrothermal liquefaction, carbonization, and gasification, respectively. The efficient conversion of unhydrolyzed residue into bio-oil, biochar, or biogas fuels enhances the overall efficiency of food waste conversion. Employing DESs or NADESs in (co)solvent-requiring or catalyst-requiring stages is believed to be a major step towards building a sustainable bioeconomy.

iii. For this type of successive two-step process, DESs or NADESs should be employed as (co)solvents or catalysts. This requires innovative design of highly efficient eutectic solvents.

## Figures and Tables

**Table 1:** Classification of DESs.

Type	General Formula	Terms
I	${\mathrm{Cat}}^{+}X^{-}z{\mathrm{MCl}}_{x}$	M = Zn, Sn, Fe, Al, Ga, In
II	${\mathrm{Cat}}^{+}X^{-}z{\mathrm{MCl}}_{x}.yH_{2}O$	M = Cr, Co, Cu, Ni, Fe
III	${\mathrm{Cat}}^{+}X^{-}z\mathrm{RZ}$	Z = CONH_2_, COOH, OH
IV	${\mathrm{MCl}}_{x}+\mathrm{RZ}={\mathrm{MCl}}_{x-1}{}^{+}.\mathrm{RZ}+{\mathrm{MCl}}_{x+1}{}^{-}$	M = Al, Zn and Z = CONH_2_
V	Non-ionic DES	Composed only of molecular substances

**Table 2:** Selected properties of some ChCl-based DESs reported in the literature.

HBD	ChCl:HBD Molar Ratio	Melting Point (°C)	Density (g cm^−3^)	Viscosity (cP)	Ref.
Ethylene glycol	0.36:0.64	−33.32			[124]
	1:2	−66	1.12	37 (25 °C)	[125,126,127]
	0.28:0.72	4.15			[124]
	1:3		1.12	19 (20 °C)	[124,127]
	1:4			19 (20 °C)	[127]
Urea	1:2	12	1.25	750 (25 °C)	[6,128]
Thiourea	1:2	69			[6]
1-methyl urea	1:2	29			[6]
1,3-dimethyl urea	1:2	70			[6]
1,1-dimethyl urea	1:2	149			[6]
Acetamide	1:2	51			[6]
Benzamide	1:2	92			[6]
Glycerol	1:1		1.16		[129]
	1:1.5				[129]
	1:2	−40	1.18	259 (25 °C)	[127,129]
	1:3		1.20	450 (20 °C)	[127]
	1:4			503 (20 °C)	[127]
CF_3_CONH_2_	1:2		1.34	77 (40 °C)	[130]
Malonic acid	1:1	10			[116]
	1:2		1.25	1124 (25 °C)	[125]
Glucose	1:1			34,400 (50 °C)	[131]
1,4-butanediol	1:3			140 (20 °C)	[127]
	1:4			88 (20 °C)	[127]
Imidazole	3:7	56		15 (70 °C)	[132]
ZnCl_2_	1:2			85,000 (25 °C)	[133]
Adipic acid	1:1	85			[116]
Benzoic acid	1:1	95			[116]
Citric acid	1:1	69			[116]
Oxalic acid	1:1	34			[116]
Phenylacetic acid	1:1	25			[116]
Phenylpropionic acid	1:1	20			[116]
Succinic acid	1:1	71			[116]
Tricarballylic acid	1:1	90			[116]

**Table 3:** DES/NADES used in biomass and food residue processes.

DES/NADES	Molar Ratio	Role of the DES/NADES	Ref.
**DES/NADES as Pretreatment Solvents**			
ChCl:Oxalic acid ChCl:Levulinic acid ChCl:Urea ChCl:Ethylene glycol ChCl:Sorbitol	1:2 1:2 1:2 1:2 1:1	Pretreatment of microalgae for solvent extraction of lipids	[110]
ChCl:Glycerol ChCl:Ethylene glycol Ethylammonium Cl:Glycerol Ethylammonium Cl:Ethylene glycol ChCl:Urea	1:2	Pretreatment media on oil palm trunk fiber	[160]
ChCl:Urea	1:2	Pretreatment for oil palm empty fruit bunch	[161]
ChCl:Ethylene glycol (under acidic condition)	1:2	Pretreatment of switchgrass to remove lignin and xylan	[26]
Ammonium thiocyanate:Urea Guanidine hydrochloride:Urea	1:2	Pretreatment for cellulose nanofibril production	[162]
ChCl:Glycerol ChCl:Urea ChCl:Imidazole	1:2 1:2 3:7	Pretreatment and saccharification of corncob residues	[84]
ChCl:Urea	1:2	Pretreatment of rice straw	[163]
ChCl:Oxalic acid ChCl:KOH ChCl:Lactic acid ChCl:Urea	1:1 and 1:2 1:4 1:2 1:2	Fractionation of waste lignocellulosic biomass and its conversion to value-added chemicals	[164]
ChCl:Lactic acid	1:10	Pretreatment to deconstruct the recalcitrant structure of eucalyptus	[165]
ChCl:Glycerol	1:2 1:3 1:6	Pretreatment of lignocellulosic date palm residues to enhance cellulose digestibility	[81]
ChCl:Ethylene glycol	1:2	Pretreatment of *eucalyptus wood globules*	[166]
ChCl:Water	Different ratios	Pretreatment and delignification of garden waste	[167]
ChCl:Urea	1:2	Pretreatment and delignification of oil palm fronds	[168]
ChCl:Glycerol	1:2	Pretreatment of lettuce leaves	[21]
ChCl:Glycerol ChCl:Ethylene glycol	1:2 1:2	Pretreatment of apple residues, potato peels, coffee silverskin, and spent brewer’s grains	[169]
ChCl:Glycerol:AlCl_3_.6H_2_O	1:2:1	Cleavage of lignin–carbohydrate complexes and the fractionation of lignin.	[80]
ChCl:Urea ChCl:Glycerol ChCl:Formic acid ChCl:Acetic acid ChCl:Oxalic acid ChCl:Malonic acid ChCl:Citric acid	1:2 1:2 1:2 1:2 1:1 1:1 1:1	Pretreatment of corn stover biomass	[8]
ChCl:Boric acid ChCl:Glycerol Betaine:Glycerol	5:2 1:1 1:1	Pretreatment of eucalyptus pulp, spruce saw dust, and wheat straw	[82]
8 ChCl-based DESs	Different ratios	Pretreatment of wood cellulose fibers	[170]
Guanidine hydrochloride:Ethylene glycol:p-toluenesulfonic acid Guanidine hydrochloride:Propylene glycol:p-toluenesulfonic acid Guanidine hydrochloride:Glycerine:p-toluenesulfonic acid ChCl:Ethylene glycol:p-toluenesulfonic acid ChCl:Propylene glycol:p-toluenesulfonic acid ChCl:Glycerine:p-toluenesulfonic acid	1:1.95:0.06 1:1.95:0.06 1:1.95:0.06 1:1.95:0.06 1:1.95:0.06 1:1.95:0.06	Pretreatment to remove lignin and xylan from switchgrass	[171]
ChCl:Urea	1:2	Pretreatment of oil palm fronds after ultrasonication in water medium	[172]
Benzyltrimethylammonium Cl:Lactic acid Benzyltriethylammonium Cl:Lactic acid	1:1 1:1	Pretreatment of corncob	[173]
ChCl:Lactic acid ChCl:Urea ChCl:Glycerol	Different ratios 1:2 1:2	Pretreatment of oil palm empty fruit bunch	[174]
ChCl with different carboxylic acids	Different ratios	Pretreatment of lignocellulosic oil palm empty fruit bunch	[175]
**DES/NADES as Extraction Solvents**			
ChCl:Urea (aqueous)	1:2	Upgrading the biogas from anaerobic digestion of biological wastes	[176]
ChCl with different monocarboxylic, dicarboxylic acids or polyalcohols	Different ratios	Delignification of corncob biomass	[22]
6 ChCl-based DESs (ChCl:Oxalic acid was the best DES)	Different ratios	Extraction of collagen peptide from cod skins	[31]
ChCl:Glycerol	1:2	Extraction of proteins from oilseed cakes	[27]
11 ChCl-based NADESs (the best one is ChCl:DL-malic acid)	Different ratios	Removing calcium carbonate and protein to produce O-acylated chitin in shrimp shells.	[177]
ChCl:Ascorbic acid	1.2:1 2:1 2.5:1	Extraction of antioxidants	[178]
Betaine monohydrate: Glycerol	1:8	Deacidification of palm oil	[179]
ChCl- or lactic acid-based DES with different HBDs	1:1	Delignification of rice straw	[180]
Various NADESs	Different ratios	Extraction of vanillin from vanilla pods	[181]
Various DESs	Different ratios	Extraction of phenolic compounds from olive oil	[32]
ChCl:Malic acid	1:1	Extraction of minerals and proteins from shrimp shells	[182]
Various DESs	1:2 or 1:1:1	Delignification and n-butanol production	[183]
Lactic acid:Glucose:Water	6:1:6	Extraction of phenolic compounds in extra virgin olive oils	[184]
Various acidic or neutral DES	Different ratios	Delignification and ethanol production	[185]
Lactic acid:Glucose Citric acid:Glucose Fructose:Citric acid	5:1 1:1 1:1	Phenolic compound extraction from agri-food byproducts	[186]
Tetrabutylammonium Cl:Decanoic acid	1:3	Extraction of quercetin from vegetable and fruit samples	[187]
ChCl:Citric acid:30% water	1:1	Extraction of isoflavones from soy products	[188]
ChCl:different HBDs	Different ratios	Extraction of anthocyanins from grape skin	[33]
Betaine:Glycerol:D-(+)-glucose	4:20:1	Extraction and storage media for bioactive natural products from green tea	[189]
ChCl:Acetic acid ChCl: Malonic acid ChCl:Citric acid	1:2 1:1 3:2	Extraction of tocols from crude palm oil	[190]
ChCl:Lactic acid Sodium acetate:Lactic acid Ammonium acetate:Lacticacid Glycine:Llactic acid:Water	3:1 3:1 3:1 3:1:3	Extraction of antioxidant polyphenols from common native Greek medicinal plants	[191]
Proline:Glycerol	2:5	Flavonoid extraction from *Flos sophorae*	[192]
Various NADESs	Different ratios	Extraction of rutin from tartary buckwheat hull	[193]
l-Proline:Glycerol	1:4	Extraction of flavonoids from *Radix scutellariae*	[194]
Various DESs	Different ratios	Extraction of different types of bioactive alkaloids	[195]
Betaine:Hexafluoroisopropanol l-Carnitine:Hexafluoroisopropanol	1:2, 1:2.5, 1:3 1:2, 1:2.5, 1:3	Microextraction of pyrethroids in tea beverages and fruit juices	[196]
ChCl:Lactic acid	1:2	Delignification of corn stover, switchgrass and Miscanthus	[197]
Various DESs	Different ratios	Extraction of bioactive flavone C-glycosides from *Flos trollii*	[198]
ChCl:Ethylene glycol	1:3	Extraction of phenolic compounds from rattan	[25]
12 ChCl-based DESs	Different ratios	Recovering polyphenols from microalgal biomass	[199]
ChCl:Lactic acid	1:1	Extraction of baicalin from *Scutellaria baicalensis* Georgi	[200]
14 ChCl-based DESs (ChCl:Malonic acid was the best DES)	1:2	Extraction of chitin from shrimp shells	[50]
ChCl:1,4–butanediol	1:5	Extraction of flavonoids from *Cyclocarya paliurus* (Batal.) Iljinskaja leaves	[29]
Various DESs	Different ratios	Extraction of hydrophilic and hydrophobic components from *Radix salviae* miltiorrhizae	[201]
ChCl:Glycerol ChCl:Oxalic acid ChCl:Malic acid ChCl:Glucose ChCl:Fructose ChCl:Xylose ChCl:Citric acid	1:2 1:1 1:1 2:1 1.9:1 2:1 Not found	Extraction of wine lees anthocyanins	[202]
ChCl:Acetic acid ChCl:Lactic acid ChCl:Levulinic acid ChCl:Glycerol	1:1	Delignification of poplar and Douglas fir wood	[203]
ChCl:Glucose ChCl:Fructose ChCl:Xylose ChCl:Glycerol ChCl:Malic acid	2:1 1.9:1 2:1 1:2 1:1	Extraction of phenolic compounds in grape skin	[204]
Various DESs	Different ratios	Extraction of alkaloids, flavonoids, saponins, anthraquinones, and phenolic acids	[111]
ChCl:Oxalic acid dihydrate ChCl:Glycerol ChCl:Urea	1:1	Delignification of poplar wood	[205]
ChCl:1,2-propanediol Lactic acid:Glucose Proline:Malic acid ChCl:Malic acid ChCl:Glucose Glucose:Fructose:Sucrose	1:1 5:1 1:1 1:1 1:1 1:1:1	Extraction of anthocyanins from *Catharanthus roseus*	[206]
7 ChCl-based DESs	1:2	Extraction of seaweed polysaccharides from *Saccharina japonica* in subcritical condition	[108]
ChCl:Malonic acid:55%Water	1:2	Extraction of proanthocyanidin from *Ginkgo biloba* leaves	[207]
Glycerol:Xylitol:D-(−)-Fructose	3:3:3	Extraction polyphenols and furanocoumarins from fig leaves	[208]
ChCl:Maltose:20% Water	1:2	Extraction and determination of phenolics in *Cajanus cajan* leaves	[209]
20 ChCl- and glycerol-based NADESs	Different ratio	Extraction of cadmium from rice flour	[210]
Various NADESs	Different ratio	Extraction of main bioactive flavonoids from *Radix ccutellariae*	[211]
ChCl:Urea ChCl:Glycerol ChCl:Thiourea ChCl:Malonic acid	1:2	Extraction of chitin from lobster shells	[212]
Various DESs	Different ratios	Extraction of saponins from sisal and juá	[213]
Glycerol:L-proline:Sucrose	9:4:1	Extraction of polar ginseng saponins from white ginseng	[214]
ChCl:Urea ChCl:Glycerol ChCl:Ethylene glycol	1:2	Extraction of k-carrageenan from *Kappaphycus alvarezii*	[215]
Lactic acid:Glucose:Water Lactic acid:Glucose Lactic acid:Glycine:Water Lactic acid:Glycine	6:1:6 and 5:1:3 5:1 3:1:1 9:1	Extraction of pectin from pomelo peels	[216]
ChCl:Phenol	1:3	Separation of caffeine from beverages	[217]
9 ChCl-based DESs (ChCl:*p*-cresol had the highest extraction efficiency)	1:2	Extraction of polar and non-polar lignans	[218]
Various DESs (ChCl:Lactic acid, 1:9, exhibits optimal extraction capacity)	Different ratios	Selective extraction of lignin from poplar wood meal	[219]
ChCl:Oxalic acid ChCl:Betaine ChCl:Urea	1.5:1 3:1 1:1	Solvent for conversion of lignocellulosic waste into HMF/furfural	[220]
ChCl:Urea ChCl:Glycerol ChCl:Citric acid ChCl:Lactic acid	1:2 1:2 1:1 1:1	Solvent for conversion of furfural into cyclopentenone derivatives	[221]
ChCl:Formic acid ChCl:Lactic acid ChCl:Acetic acid Betaine:Lactic acid Proline:Lactic acid	1:2 1:10 1:2 1:2 1:3.3	Solvents to solubilize lignocellulosic components	[222]
ChCl:Oxalic acid ChCl:Citric acid ChCl:Tartaric acid	1:1 0.7:0.3 0.7:0.3	Solvent and catalyst for conversion of cellulose into low molecular compounds	[223]
Various NADESs	Different ratios	Solvent to solubilize proteins	[224]
ChCl:Imidazole Imidazole:Glycerol Imidazole:Citric acid Imidazole:Malic acid	3:7 and 2:3 1:1 and 7:3 7:3 7:3	Starch dissolution and plasticization	[225]
ChCl:Lactic acid	1:2	Extraction of lignin nanoparticles from wheat straw	[226]
ChCl:Levulinic acid:Methyl urea	1:1:1	Extraction of flavonoids from citrus peel waste	[227]
11 ChCl-based DESs	1:1 to 1:3	Extract bioactive compounds from *Lycium barbarum* L. fruits	[228]
**DES/NADES as Catalyst**			
ChCl:KOH ChCl:*p*-Toluenesulfonic acid monohydrate ChCl:Glycerol ChCl:FeCl_3_	1:4 1:4 1:3 1:3	Catalyst and cosolvent for hydrothermal liquefaction of de-oiled *Jatropha curcas* cake	[24]
ChCl:*p*-Toluenesulphonic acid	1:3, 1:5, 1:7	Catalyst in co-liquefaction of *Jatropha curcas* seed	[23]
Citric acid:Alanine	1:1	Catalyst in extraction of phenolic compounds from mangosteen pericarps in subcritical water	[109]
ChCl:Oxalic acid	Different ratios	Conversion of biomass furfural to fumaric acid and maleic acid in the presence of H_2_O_2_	[229]
ChCl:*p*-Toluenesulfonic acid	1:6	Using DES as heterogeneous and homogeneous catalysts to produce biodiesel from *Pongamia pinnata* seed oil	[230]
